# Supplementary material for: Synthesis and Preclinical Evaluation of Radiolabeled [103Ru]BOLD-100
Source: Pharmaceutics. 2023 Nov 15;15(11):2626. doi: 10.3390/pharmaceutics15112626 (PMC10674160; doi:10.3390/pharmaceutics15112626)
Supplement: Supplementary file 1 [file pharmaceutics-15-02626-s001.zip › pharmaceutics-2647681-supplementary.pdf]

Supporting information

Synthesis and preclinical evaluation of radiolabeled [^{103}Ru]BOLD-100

B. Happl,[1,2,3] M. Brandt,[1,2,4] T. Balber,[1,2,4] K. Benčurová,[1,2] Z. Talip,[5] A. Voegele,[6] P. Heffeter,[7,8] W. Kandioller,[3,8] N. P. van der Meulen,[5,6] M. Mitterhauser,[1,2,3,4] M. Hacker,[2] B. K. Keppler,[3,8] and T. L. Mindt[1,2,3,4]*

[1] Ludwig Boltzmann Institute Applied Diagnostics, General Hospital of Vienna, Waehringer Guertel 18-20, 1090 Vienna, Austria

[2] Division of Nuclear Medicine, Department of Biomedical Imaging and Image Guided Therapy, Medical University of Vienna, Waehringer Guertel 18-20, 1090 Vienna, Austria

[3] Institute of Inorganic Chemistry, Faculty of Chemistry, University of Vienna, Waehringer Strasse 42, 1090 Vienna, Austria

[4] Joint Applied Medicinal Radiochemistry Facility, University of Vienna, Medical University of Vienna

[5] Center for Radiopharmaceutical Sciences, Paul Scherrer Institute, Forschungsstrasse 111, 5232 Villigen, Switzerland

[6] Laboratory of Radiochemistry, Paul Scherrer Institute, Forschungsstrasse 111, 5232 Villigen, Switzerland

[7] Institute of Cancer Research, Comprehensive Cancer Center, Medical University of Vienna, Borschkegasse 8A, 1090 Vienna, Austria

[8] Research Cluster "Translational Cancer Therapy Research", Waehringer Strasse 42, 1090 Vienna, Austria

Table of contents

1. ICP-MS parameter 3
2. Gamma spectrum 5
3. UV/Vis spectra 6
4. HPLC[1] 7
5. Cell viability curves 11
6. Biodistribution data 12
7. References 16

1. ICP-MS parameter

An Agilent 7800 ICP-MS (Agilent Technologies, Tokyo, Japan) was being employed, which was equipped with an Agilent SPS 4 autosampler (Agilent Technologies, Tokyo, Japan) and a MicroMist nebulizer at a sample uptake rate of approx. 0.2 mL/min. The Agilent MassHunter software package (Workstation Software, Version C.01.04, 2018) was used for data evaluation. The instrumental parameters for the ICP-MS are summarized in **Table S1**Error! Reference source not found..

Table S1: Instrumental parameters for the ICP-MS measurements

RF power	1550 W
Sampling depth	6.3 mm
Nebulizer	MicroMist
Spray chamber	Scott double-pass
Spraying chamber temp.	2°C
Monitored Isotopes	^{101}Ru, ^{102}Ru, ^{115}In
Measurement mode	no gas
Plasma gas	15 L min^{-1}
Nebulizer gas	1.06 L min^{-1}
Auxiliary gas	0.90 L min^{-1}
Cones	Ni
Cell entrance	-60 V
Cell exit	-110 V
Integration time	0.1 s
Replicates	10

The instrumental limit of quantification (LOQ) was determined at the start of the measurement by measuring a blank (3% HNO3) 5 times and was found to be 4.3 ng/L.

$$LOQ = \text{blank average} + 10*STD$$

Blank corrections were performed for all the samples. Robustness was ensured by tuning the ICP-MS on a daily basis, by using an internal standard (^{115}In) and measuring calibration standards for each measurement.

Chemicals and reagents

Ultrapure water (18.2 MΩ cm, ELGA Water purification system, Purelab Ultra MK 2, UK or 18.2 MΩ cm, Milli-Q Advantage, Darmstadt, Germany), HNO_3 (≥69%, Rotipuran Supra, Carl Roth, Karlsruhe, Germany) and H_2O_2 (30%, Suprapur, Merck, Darmstadt, Germany) were used for all dilutions for ICP-MS measurements. The Ru standard solution was purchased from Labkings (Hilversum, The Netherlands).

Digestion of the samples

For the digestion, approx. 25–50 mg of each organ sample were weighed into PFA-tubes, with 2 mL HNO_3 and 100 µL H_2O_2 being added. The solutions were then placed on a hot plate and heated up for 6 hours using a temperature program with a maximum temperature of 200 °C. After cooling down, the liquids were transferred directly to 15 mL tubes with the remaining solution being removed from the PFA tubes by washing twice with 4 mL ultrapure water, resulting in a final volume of about 10 mL. By writing down the total weight of the resulting liquid, the dilution factor could be calculated.

2. Gamma spectrum

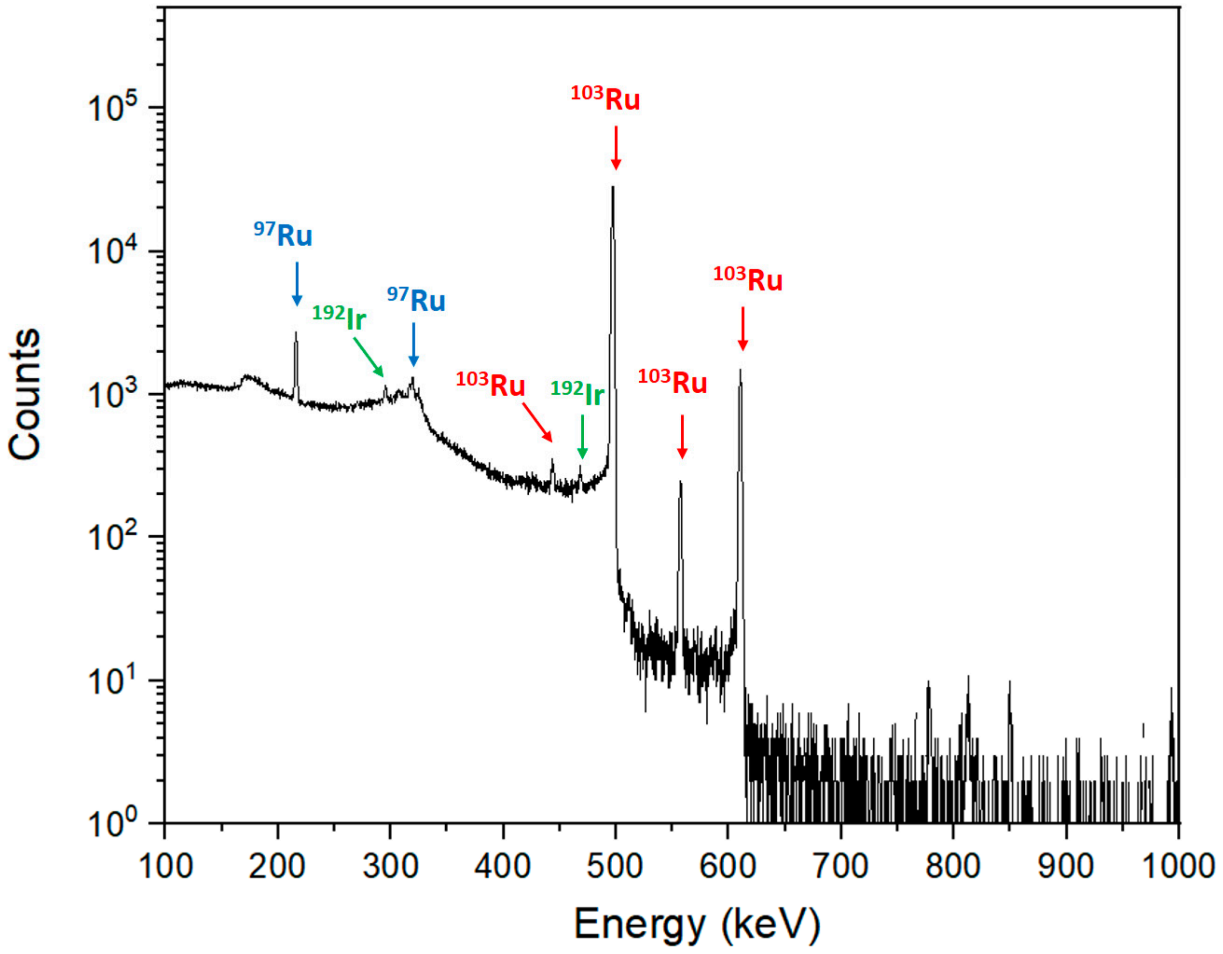

Figure S1: Representative γ-ray spectrum of [^{103}Ru]RuCl$_3$. Due to the irradiation of natural Ru, traces of ^{97}Ru ($t_{1/2}$ = 2.9 d) as well as ^{192}Ir ($t_{1/2}$ = 73.83 d) were observed.

3. UV/Vis spectra

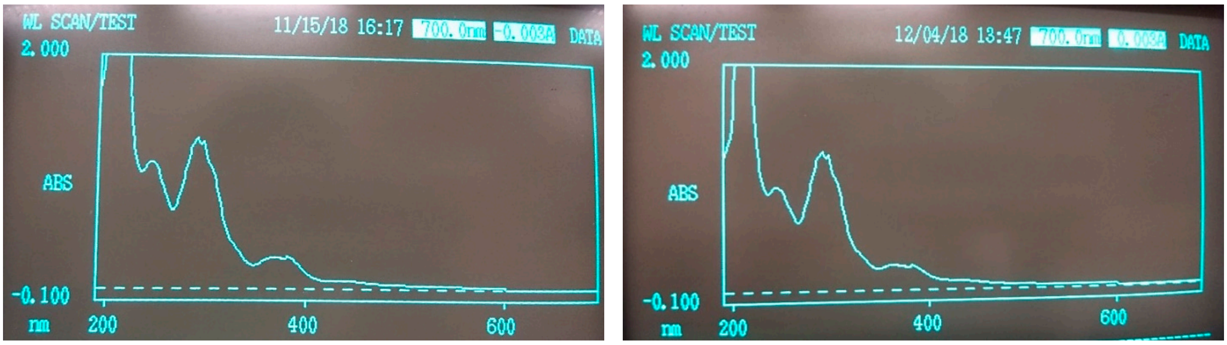

Figure S2: UV/Vis spectrum of **1a** (left, 0.068 mM) and **1b** (right, 0.059 mM), λ_{max} = ~300 nm

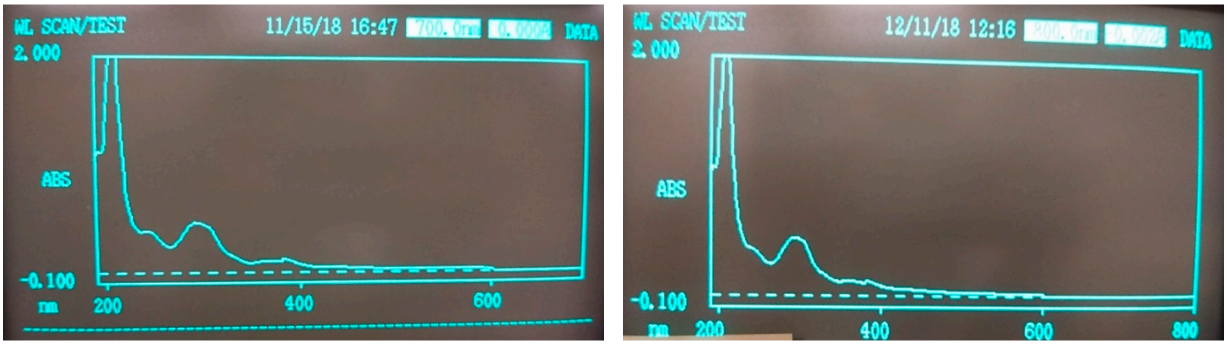

Figure S3: UV/Vis spectrum of **2a** (left, 0.027 mM) and **2b** (right, 0.035 mM), λ_{max} = ~300 nm

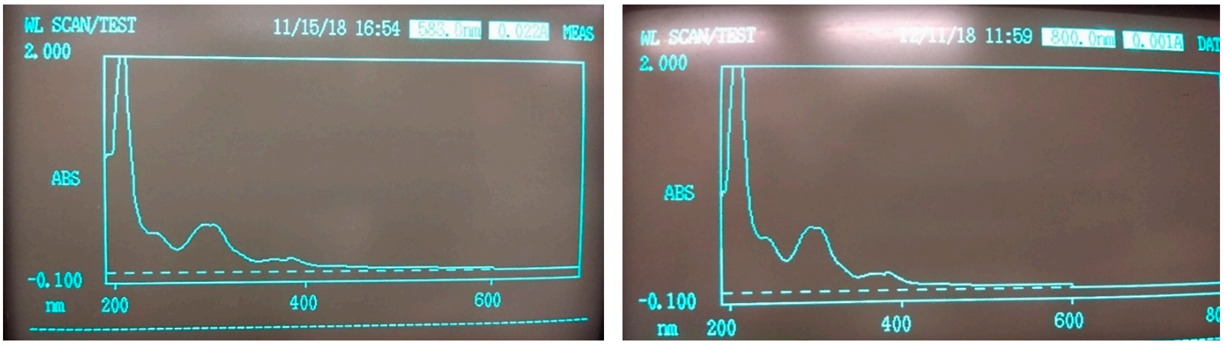

Figure S4: UV/Vis spectrum of **3a** (left, 0.025 mM) and **3b** (right, 0.035 mM), λ_{max} = ~300 nm

4. HPLC[1]

All impurities resulted from the dissolution of the samples in the HPLC starting conditions (10% ACN + 0.1% TFA, 90% H_2O + 0.1% TFA).

4.1 UV chromatograms of non-radioactive complexes

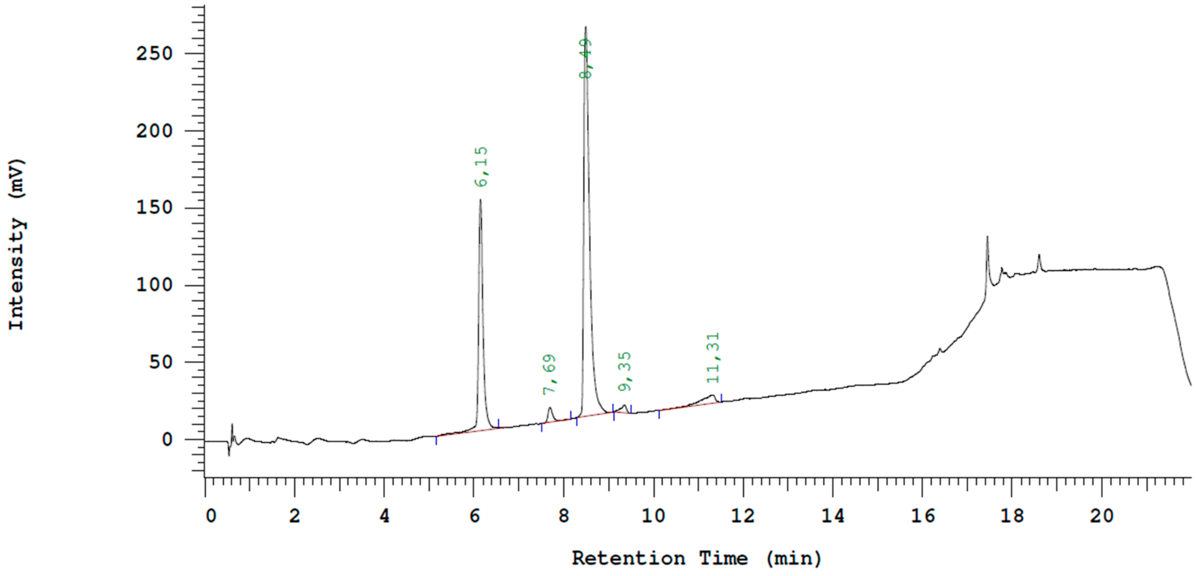

Figure S5: UV chromatogram of **1a** (220 nm, 20 µL injection, manually integrated); t_R ($Hind^+$) = 6.15 min, t_R ($[Ru(ind)_2Cl_4]^-$) = 8.49 min, t_R ($[Ru(ind)_2Cl_3(H_2O)]$) = 9.35 min, t_R ($[Ru(ind)_2Cl_3(ACN)]$) = 11.31 min.

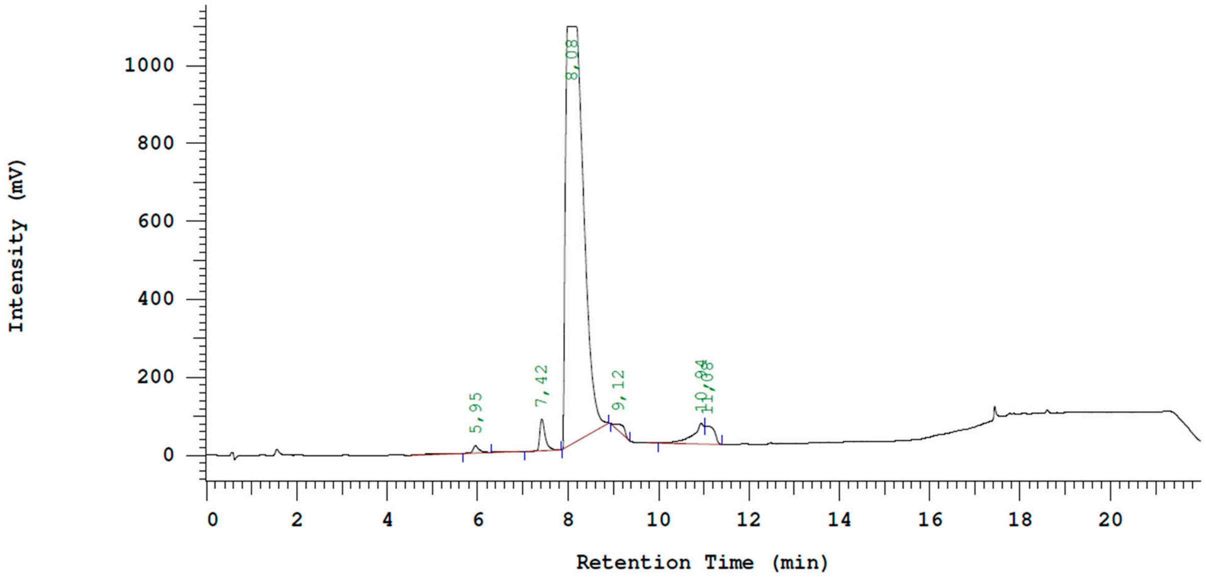

Figure S6: UV chromatogram of **2a** (220 nm, 20 µL injection, manually integrated); t_R ($[Ru(ind)_2Cl_4]^-$) = 8.08 min, t_R ($[Ru(ind)_2Cl_3(H_2O)]$) = 9.12 min, t_R ($[Ru(ind)_2Cl_3(ACN)]$) = 10.94, 11.08 min.

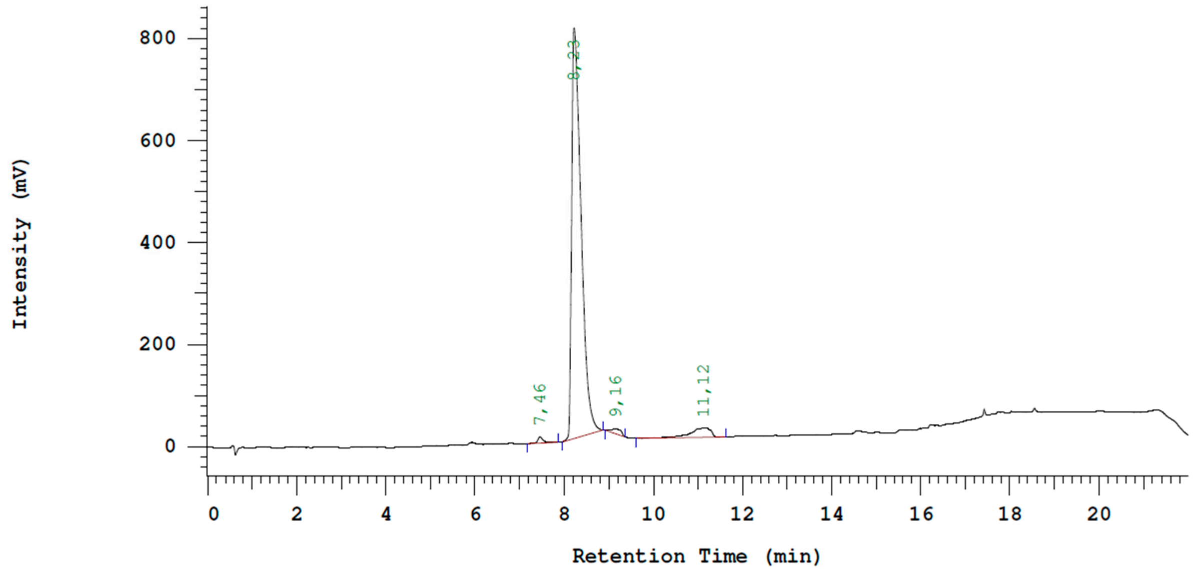

Figure S7: UV chromatogram of **3a** (220 nm, 20 µL injection, manually integrated); t_R ($[Ru(ind)_2Cl_4]^-$) = 8.23 min, t_R ($[Ru(ind)_2Cl_3(H_2O)]$) = 9.16 min, t_R ($[Ru(ind)_2Cl_3(ACN)]$) = 11.12 min.

4.2 Chromatograms of radiolabeled complexes

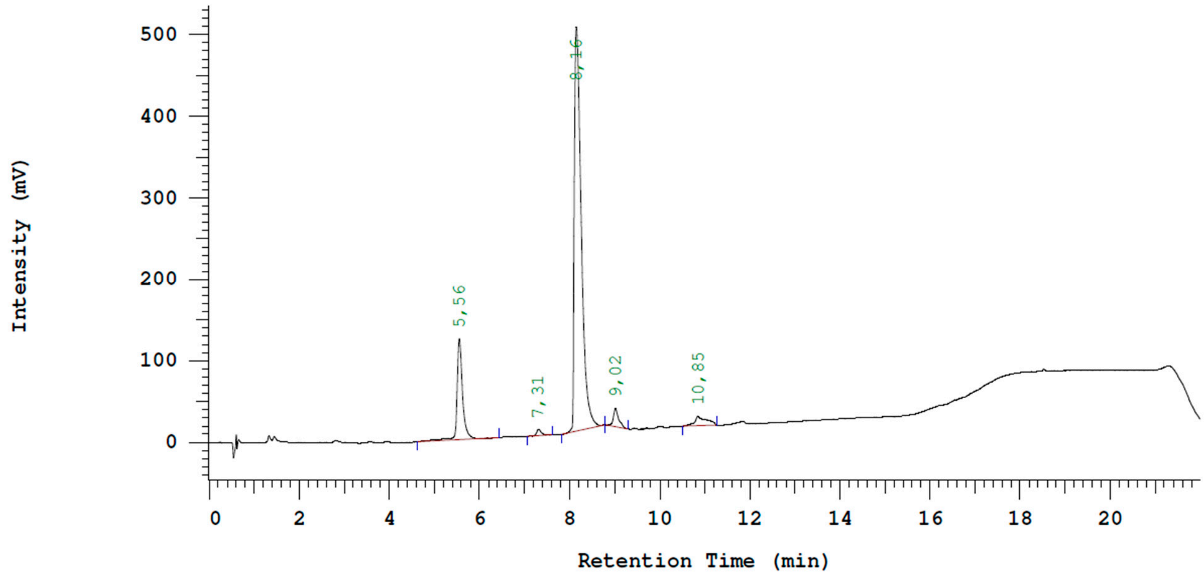

Figure S8: UV chromatogram of **1b** (220 nm, 20 µL injection, manually integrated); t_R ($Hind^+$) = 5.56 min, t_R ($[^{103}Ru][Ru(ind)_2Cl_4]^-$) = 8.16 min, t_R ($[^{103}Ru][Ru(ind)_2Cl_3(H_2O)]$) = 9.02 min, t_R ($[^{103}Ru][Ru(ind)_2Cl_3(ACN)]$) = 10.85 min.

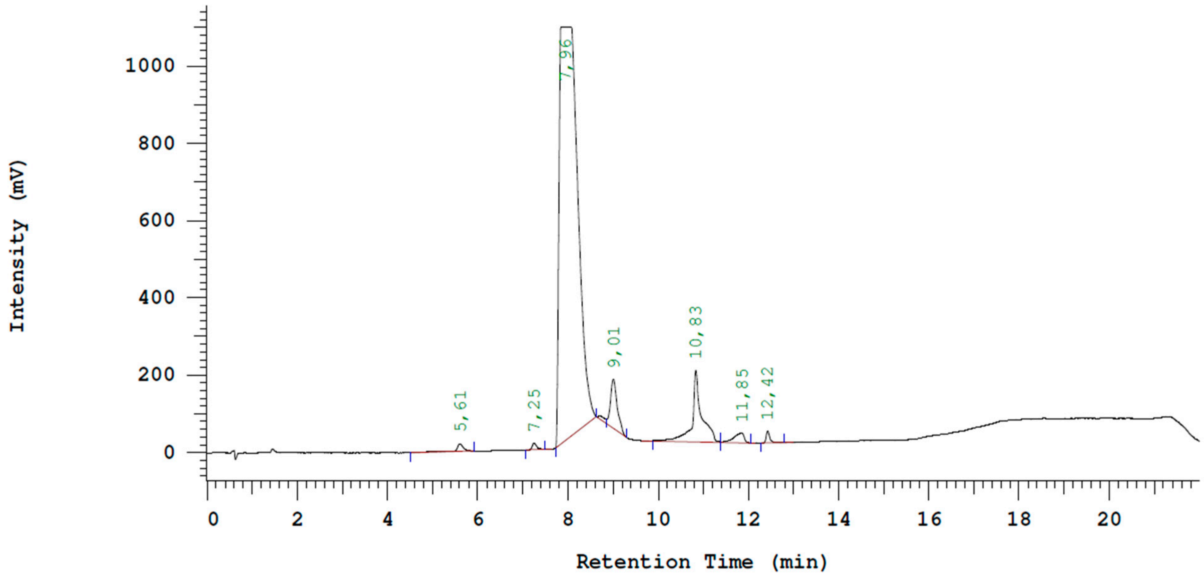

Figure S9: UV chromatogram of **2b** (220 nm, 20 µL injection, manually integrated); t_R ([^{103}Ru][Ru(ind)$_2$Cl$_4$]$^-$) = 7.96 min, t_R ([^{103}Ru][Ru(ind)$_2$Cl$_3$(H$_2$O)]) = 9.01 min, t_R ([^{103}Ru][Ru(ind)$_2$Cl$_3$(ACN)]) = 10.83 min.

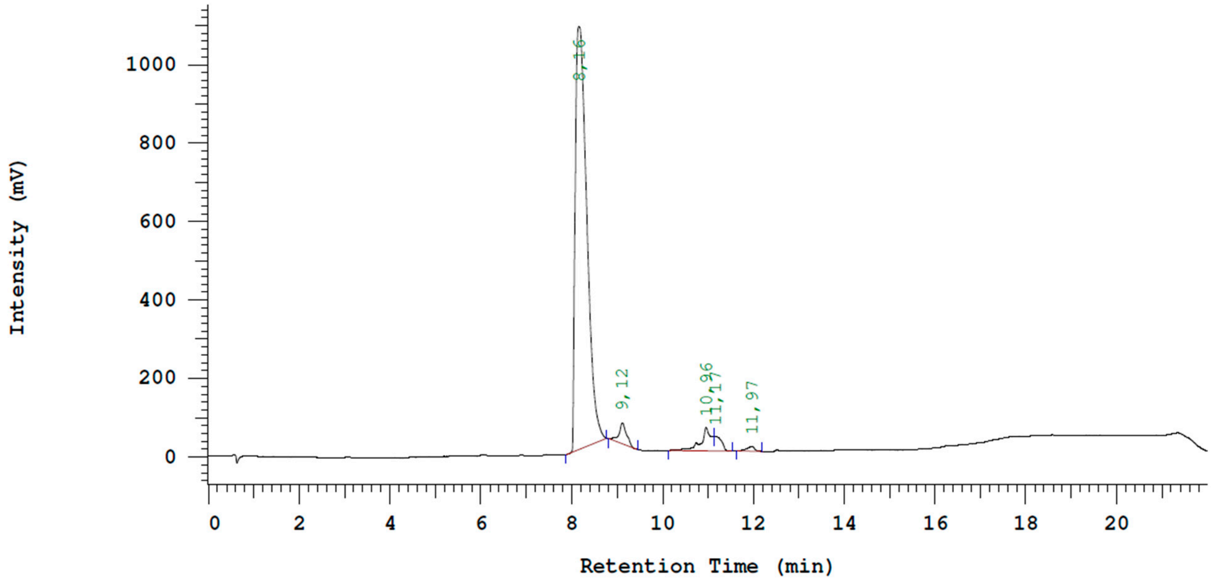

Figure S10: UV chromatogram of **3b** (220 nm, 20 µL injection, manually integrated); t_R ([^{103}Ru][Ru(ind)$_2$Cl$_4$]$^-$) = 8.16 min, t_R ([^{103}Ru][Ru(ind)$_2$Cl$_3$(H$_2$O)]) = 9.12 min, t_R ([^{103}Ru][Ru(ind)$_2$Cl$_3$(ACN)]) = 10.96, 11.17 min.

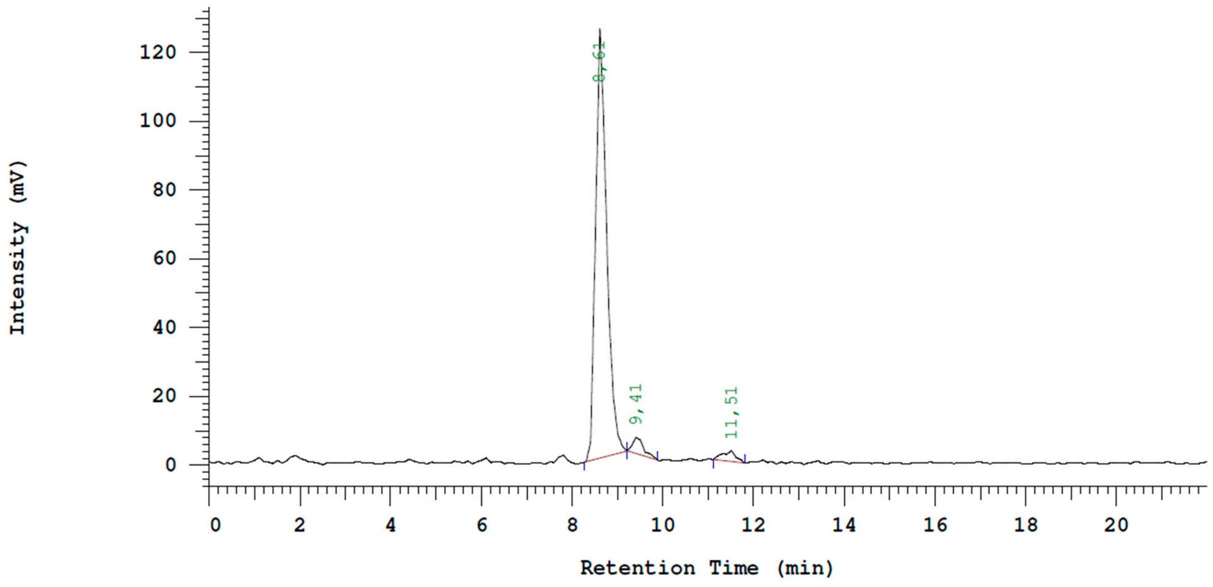

Figure S11: Radio chromatogram of **1b** (20 µL injection, manually integrated); t_R ([^{103}Ru][Ru(ind)$_2$Cl$_4$]$^-$) = 8.61 min, t_R ([^{103}Ru][Ru(ind)$_2$Cl$_3$(H$_2$O)]) = 9.41 min, t_R ([^{103}Ru][Ru(ind)$_2$Cl$_3$(ACN)]) = 11.51 min.

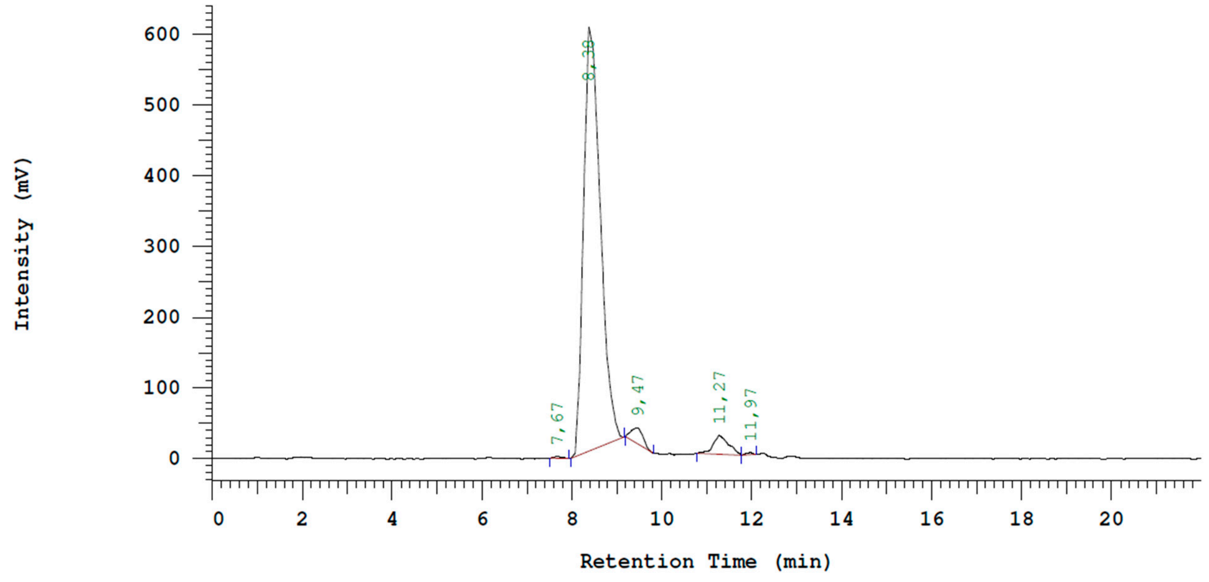

Figure S12: Radio chromatogram of **2b** (20 µL injection, manually integrated); t_R ([^{103}Ru][Ru(ind)$_2$Cl$_4$]$^-$) = 8.38 min, t_R ([^{103}Ru][Ru(ind)$_2$Cl$_3$(H$_2$O)]) = 9.47 min, t_R ([^{103}Ru][Ru(ind)$_2$Cl$_3$(ACN)]) = 11.27 min.

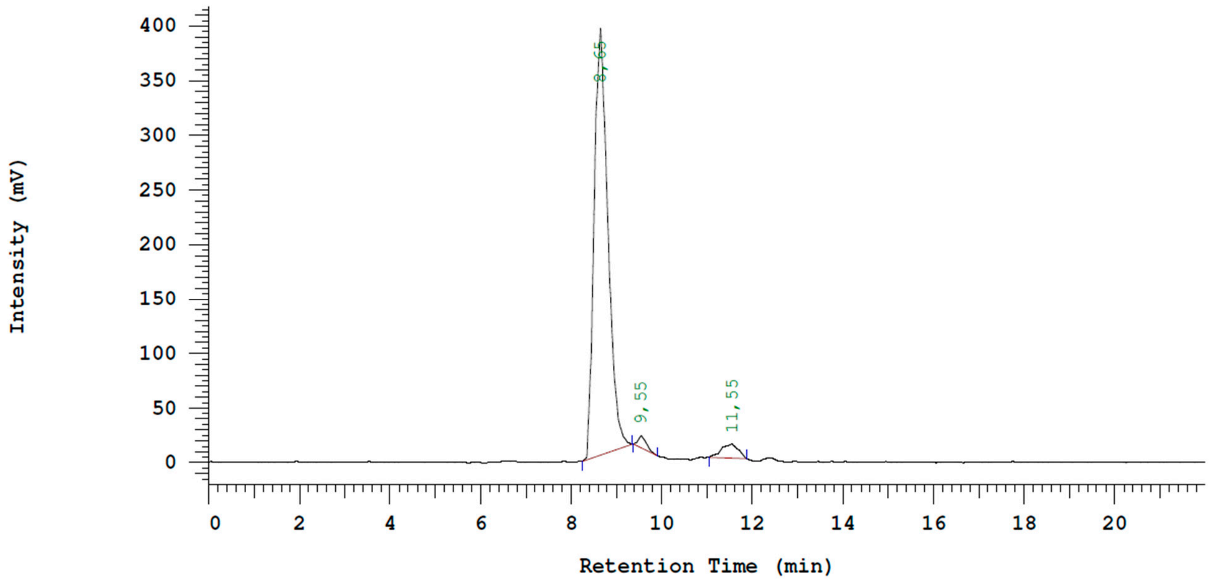

Figure S13: Radio chromatogram of **3b** (20 µL injection, manually integrated); t_R ([^{103}Ru][Ru(ind)$_2$Cl$_4$]$^-$) = 8.65 min, t_R ([^{103}Ru][Ru(ind)$_2$Cl$_3$(H$_2$O)]) = 9.55 min, t_R ([^{103}Ru][Ru(ind)$_2$Cl$_3$(ACN)]) = 11.55 min.

5. Cell viability curves

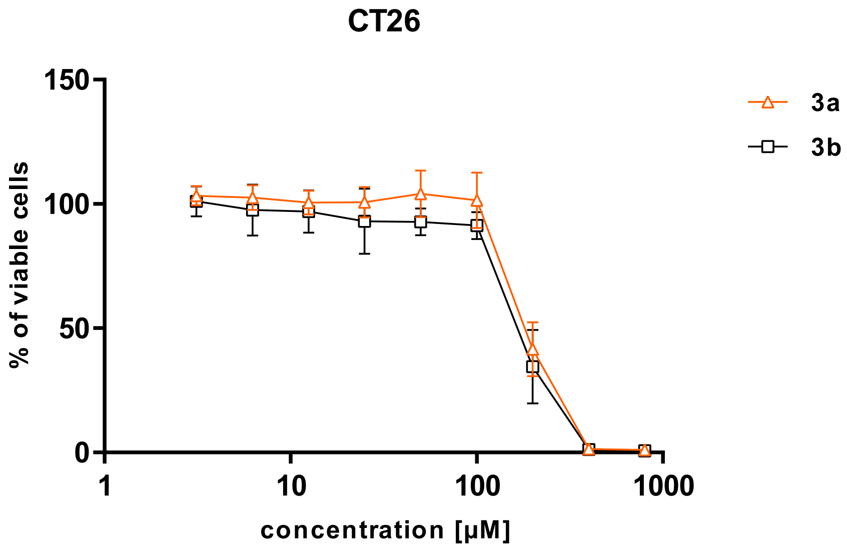

Figure S14: Cytotoxicity of **3a** (BOLD-100) and **3b** ([^{103}Ru]BOLD-100) in CT26 cell line. Values are mean ± standard deviations of n = 5 (**3a**) and n = 3 (**3b**) representative experiments performed in triplicates.

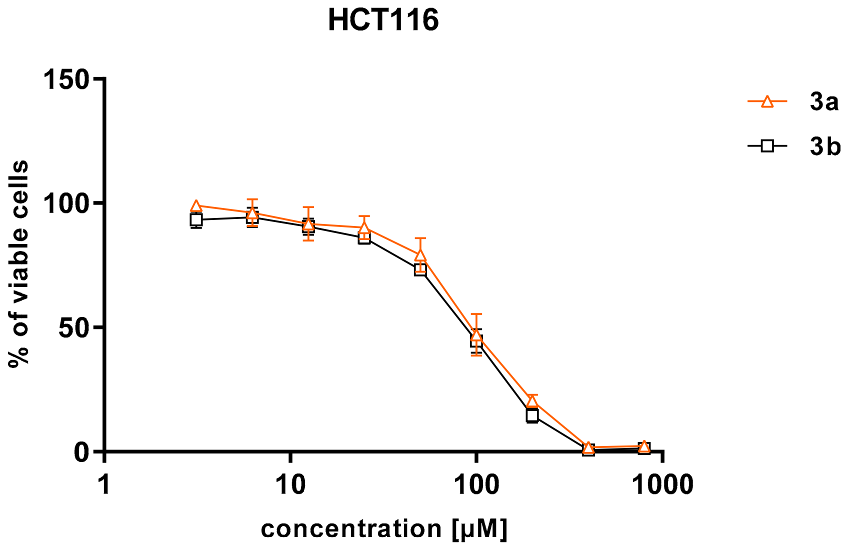

Figure S15: Cytotoxicity of **3a** (BOLD-100) and **3b** ([^{103}Ru]BOLD-100) HCT116 cell line. Values are mean ± standard deviations of n = 6 (**3a**) and n = 3 (**3b**) representative experiments performed in triplicates.

6. Biodistribution data

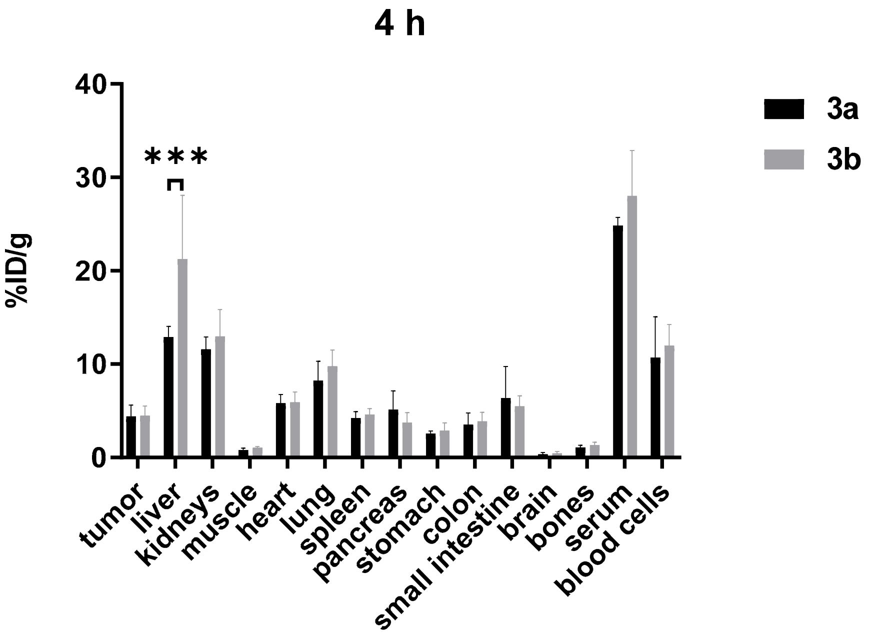

Figure S16: Tissue distribution of **3a** (BOLD-100) and **3b** ([^{103}Ru]BOLD-100) 4 h p.i. (%ID/g = percent injected dose of $^{nat/103}$Ru per gram tissue). Values are means, error bars represent standard deviations of n ≥ 2 (**3a**) and n ≥ 4 (**3b**). Comparison of data was performed by using a two-way ANOVA with Sidak's correction. Values of $p < 0.05$ were considered as statistically significant (*** $p \leq 0.001$).

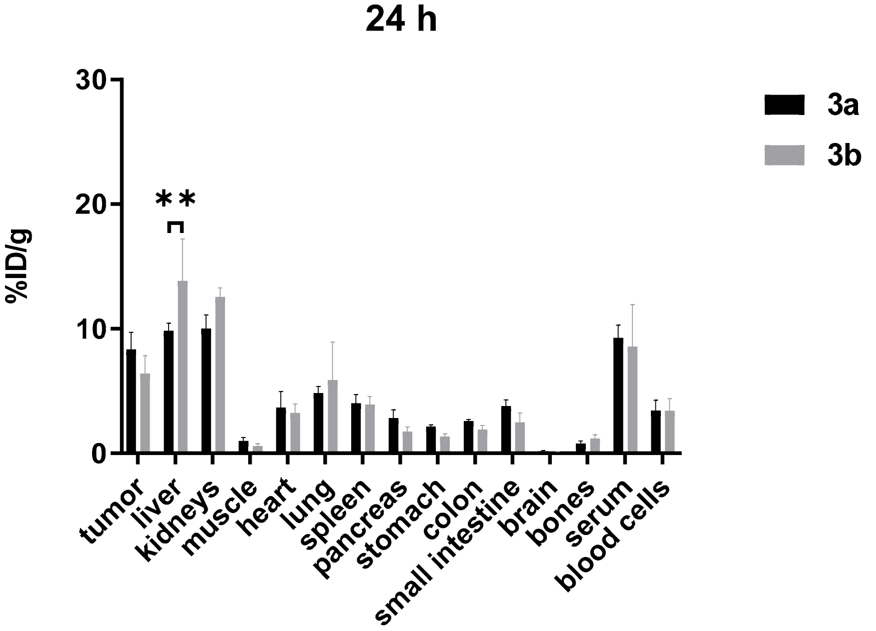

Figure S17: Tissue distribution of **3a** (BOLD-100) and **3b** ([^{103}Ru]BOLD-100) 24 h p.i. (%ID/g = percent injected dose of $^{nat/103}$Ru per gram tissue). Values are means, error bars represent standard deviations of n = 3 (**3a**) and n = 4 (**3b**). Comparison of data was performed by using a two-way ANOVA with Sidak's correction. Values of $p < 0.05$ were considered as statistically significant (** $p \leq 0.01$).

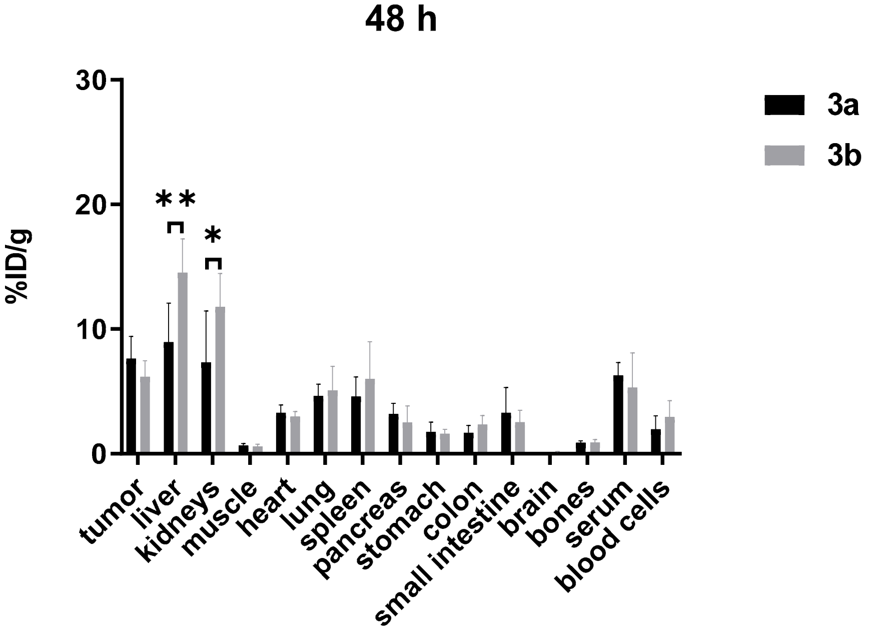

Figure S18: Tissue distribution of **3a** (BOLD-100) and **3b** ([^{103}Ru]BOLD-100) 48 h p.i. (%ID/g = percent injected dose of $^{nat/103}$Ru per gram tissue). Values are means, error bars represent standard deviations of n ≥ 2

(**3a**) and n $\geq$ 3 (**3b**). Comparison of data was performed by using a two-way ANOVA with Sidak's correction. Values of $p < 0.05$ were considered as statistically significant (* $p \leq 0.05$, ** $p \leq 0.01$).

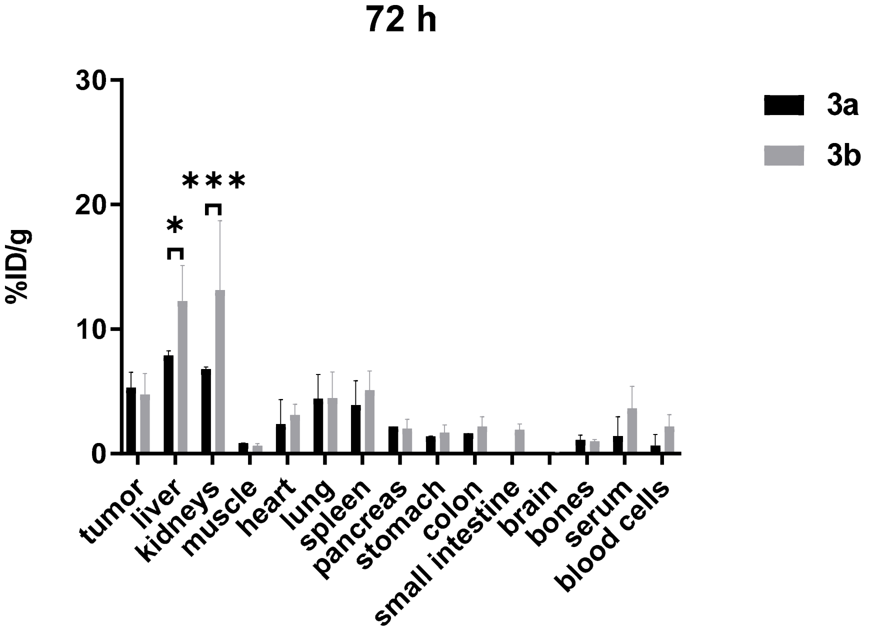

Figure S19: Tissue distribution of **3a** (BOLD-100) and **3b** ([^{103}Ru]BOLD-100) 72 h p.i. (%ID/g = percent injected dose of $^{nat/103}$Ru per gram tissue). Values are means, error bars represent standard deviations of n = 2 (**3a**) and n = 8 (**3b**). Comparison of data was performed by using a two-way ANOVA with Sidak's correction. Values of $p < 0.05$ were considered as statistically significant (* $p \leq 0.05$, *** $p \leq 0.001$). Data of **3a** for small intestine could not be obtained, due to raw data inconsistency.

Table S2: Tissue distribution data of **3a** (BOLD-100) and **3b** ([^{103}Ru]BOLD-100) in CT26 bearing Balb/c mice over 72 h. Values are means ± standard deviations of n ≥ 2 (**3a**) and n ≥ 3 (**3b**). %ID/g = percent injected dose of $^{nat/103}$Ru per gram tissue.

Tissue	%ID/g tissue			
	4 h		24 h	
	**3a**	**3b**	**3a**	**3b**
Tumor	4.43 ± 1.20	4.49 ± 1.03	8.34 ± 1.36	6.39 ± 1.44
Liver	12.90 ± 1.14	24.41 ± 3.17	9.84 ± 0.60	13.83 ± 3.38
Kidneys	11.58 ± 1.35	12.98 ± 2.87	10.02 ± 1.09	12.55 ± 0.74
Muscle	0.82 ± 0.18	1.05 ± 0.14	0.99 ± 0.28	0.57 ± 0.19
Heart	5.81 ± 0.94	5.93 ± 1.10	3.66 ± 1.31	3.24 ± 0.73
Lung	8.24 ± 2.06	9.77 ± 1.75	4.84 ± 0.53	5.89 ± 3.04
Spleen	4.23 ± 0.68	4.63 ± 0.61	4.02 ± 0.71	3.92 ± 0.66
Pancreas	5.14 ± 2.01	3.75 ± 1.07	2.81 ± 0.68	1.74 ± 0.39
Stomach	2.57 ± 0.29	2.88 ± 0.82	2.13 ± 0.17	1.34 ± 0.22
Colon	3.53 ± 1.27	3.89 ± 0.96	2.58 ± 0.13	1.88 ± 0.37
Small intestine	6.38 ± 3.36	5.49 ± 1.12	3.78 ± 0.51	2.49 ± 0.76
Brain	0.36 ± 0.19	0.46 ± 0.20	0.15 ± 0.07	0.12 ± 0.02
Bones	1.08 ± 0.25	1.34 ± 0.30	0.79 ± 0.20	1.19 ± 0.29
Serum	24.85 ± 0.85	28.02 ± 4.87	9.26 ± 1.03	8.57 ± 3.37
Blood cells	10.70 ± 4.38	11.98 ± 2.26	3.43 ± 0.83	3.41 ± 0.97

Tissue	%ID/g tissue			
	48 h		72 h	
	**3a**	**3b**	**3a**	**3b**
Tumor	7.64 ± 1.78	6.19 ± 1.26	5.32 ± 1.22	4.77 ± 1.68
Liver	8.96 ± 3.11	14.54 ± 2.69	7.90 ± 0.35	12.26 ± 2.85
Kidneys	7.34 ± 4.13	11.79 ± 2.66	6.78 ± 0.18	13.14 ± 5.56
Muscle	0.66 ± 0.16	0.60 ± 0.16	0.84 ± 0.02	0.65 ± 0.17
Heart	3.28 ± 0.63	2.98 ± 0.41	2.37 ± 1.97	3.12 ± 0.84
Lung	4.64 ± 0.94	5.08 ± 1.93	4.41 ± 1.94	4.47 ± 2.10
Spleen	4.59 ± 1.57	6.01 ± 2.98	3.92 ± 1.93	5.08 ± 1.56
Pancreas	3.18 ± 0.85	2.50 ± 1.33	2.19 ± 0.01	2.01 ± 0.75
Stomach	1.76 ± 0.77	1.60 ± 0.36	1.40 ± 0.01	1.69 ± 0.64
Colon	1.68 ± 0.58	2.34 ± 0.72	1.63 ± 0.00	2.19 ± 0.77
Small intestine	3.28 ± 2.02	2.54 ± 0.95	0.00 ± 0.00	1.92 ± 0.47
Brain	0.10 ± 0.02	0.13 ± 0.05	0.11 ± 0.00	0.09 ± 0.05
Bones	0.88 ± 0.16	0.90 ± 0.24	1.12 ± 0.35	1.00 ± 0.15
Serum	6.29 ± 1.02	5.31 ± 2.78	1.41 ± 1.56	3.63 ± 1.78
Blood cells	1.96 ± 1.09	2.94 ± 1.32	0.67 ± 0.86	2.19 ± 0.96

Table S3: Tumor-to-organ ratio of **3b** ([^{103}Ru]BOLD-100). Values are means ± standard deviations.

Organ	tumor/organ ratio of [^{103}Ru]BOLD-100 (**3b**)			
	**4 h**	**24 h**	**48 h**	**72 h**
Liver	0.2 ± 0.2	0.5 ± 0.1	0.4 ± 0.1	0.4 ± 0.1
Kidneys	0.4 ± 0.1	0.5 ± 0.1	0.5 ± 0.1	0.4 ± 0.2
Muscle	4.4 ± 1.4	11.4 ± 1.8	10.6 ± 1.4	7.6 ± 2.4
Heart	0.8 ± 0.2	2.0 ± 0.2	2.1 ± 0.2	1.5 ± 0.4
Lung	0.5 ± 0.2	1.3 ± 0.5	1.3 ± 0.3	1.2 ± 0.4
Spleen	1.0 ± 0.2	1.7 ± 0.3	1.1 ± 0.3	1.0 ± 0.3
Pancreas	1.2 ± 0.3	3.7 ± 0.7	2.7 ± 0.7	2.6 ± 0.9
Stomach	1.7 ± 0.7	4.8 ± 1.2	3.9 ± 0.3	3.0 ± 1.0
Fat	5.1 ± 2.1	10.3 ± 2.1	13.3 ± 5.0	8.6 ± 3.5
Colon	1.2 ± 0.6	3.4 ± 0.7	2.8 ± 0.8	2.3 ± 0.8
Small intestine	0.9 ± 0.3	2.8 ± 1.1	2.6 ± 0.8	2.6 ± 0.9
Brain	10.7 ± 3.3	51.9 ± 4.4	53.3 ± 14.3	54.3 ± 15.1
Bones	3.4 ± 0.5	5.4 ± 0.6	7.0 ± 0.8	4.9 ± 1.7
Serum	0.2 ± 0.0	0.8 ± 0.1	1.3 ± 0.4	1.4 ± 0.3
Blood cells	0.4 ± 0.1	2.0 ± 0.6	2.3 ± 0.6	2.3 ± 0.8

7. References

1. Vojkovsky, T.; Sill, K.; Carie, A. Manufacture of trans-[tetrachlorobis(1h-indazole)ruthenate (iii)] and compositions thereof, *WO2018204930A1*, 08.11.2018.
